# Genetic correlation between Prothrombin G20210A polymorphism and retinal vein occlusion risk

**DOI:** 10.1590/1414-431X20198217

**Published:** 2019-04-08

**Authors:** Yuanyuan Zou, Xi Zhang, Jingyi Zhang, Xiangning Ji, Yuqing Liu, Shaozhen Zhao

**Affiliations:** 1 Department of Refraction and Cornea, Tianjin Medical University Eye Hospital, School of Optometry and Ophthalmology, Tianjin Medical University, Tianjin, China; 2 The Second Department of Ophthalmology, Cangzhou Central Hospital, Cangzhou, China

**Keywords:** Prothrombin, Retinal vein occlusion, Polymorphism, Risk, Meta-analysis

## Abstract

The aim of this study was to perform an updated meta-analysis to quantitatively investigate the association between G20210A polymorphism of Prothrombin gene and the risk of retinal vein occlusion (RVO), based on the available publications with inconsistent results. We utilized the Stata software to perform the heterogeneity test, association test, Begg's and Egger's tests, and sensitivity analysis. We searched three on-line databases (PubMed, Embase, and WOS) and obtained a total of 422 articles. Based on our selection criteria, 24 case-control studies were finally enrolled in this overall meta-analysis; a subgroup analysis by the factors ethnicity, control source, and RVO type was done. Through the association test of overall meta-analysis, we did not observe a significant difference between RVO cases and controls under the A *vs* G (allele) (z=1.49, P=0.137), A *vs* G (carrier) (z=1.42, P =0.155), GA *vs* GG (z=1.50, P=0.135), and GA+AA *vs* GG (z=1.50, P=0.135). Furthermore, we observed similar negative results in the association test of subgroup analysis (all P>0.05). Heterogeneity, Begg's, and Egger's tests excluded the presence of high heterogeneity and publication bias. Statistically stable results were observed in the sensitivity analyses. Based on integrated analysis of the current evidence, Prothrombin gene G20210A polymorphism is likely unrelated to the risk of RVO.

## Introduction

Retinal vein occlusion (RVO) is a common retinal vascular disease, and often contributes to the occurrence of visual decline or loss, especially for middle-aged or elderly individuals (1). The main clinical characteristics of RVO include retinal vein dilatation, retinal and subretinal hemorrhages, macular edema, or retinal ischemia (1). Central retinal vein occlusion (CRVO) and branch retinal vein occlusion (BRVO) are two main types of RVO (1,2). The exact pathogenesis of RVO remains unclear. Genetic variants within a series of genes were reportedly associated with the risk of RVO (3).

Factor V G1691A (Factor V Leiden or R506Q) and G20210A polymorphism (rs1799963) within Prothrombin (Factor II) gene are the most common inherited thrombophilic mutations (4). Previously, we conducted an updated meta-analysis and reported that “GA” genotype of Factor V G1691A polymorphism is associated with an increased susceptibility to RVO (particularly CRVO) in a Caucasian population (2). Herein, we investigated the role of Prothrombin G20210A polymorphism in the risk of RVO. Prothrombin G20210A polymorphism may lead to the alteration of a single base from guanine (G) to adenine (A) at “20210” site in the 3′-untranslated region, and the impaired enzyme activity of prothrombin protein.

To the best of our knowledge, only two meta-analyses on the genetic role of Prothrombin G20210A in the susceptibility to RVO were reported in 2005 (5) and 2013 (6). In the present study, a total of 24 eligible case-control studies were enrolled for our updated meta-analysis, which followed the preferred reporting items for systematic reviews and meta-analyses (PRISMA) (2).

## Material and Methods

### Database search

Three authors (Y. Zou, X. Zhang, and J. Zhang) performed the on-line database search (updated to November 2018) to obtain the related published articles. Three on-line databases, including PubMed, Excerpta Medica Database (Embase), and Web of Science (WOS), were electronically searched. No restrictions of publication period or language were utilized. Detailed search terms are shown in Supplementary Table S1.

### Selection strategy

Three authors (Y. Zou, X. Ji, and Y. Liu) selected the eligible case-control studies. Based on the principles of PICOS (population, intervention, comparator, outcomes and study designs), the specific inclusion criteria were utilized: (P) cases of RVO; (I) Prothrombin G20210A polymorphism; (C) healthy individuals or negative controls; the genotype frequency distribution should follow Hardy-Weinberg equilibrium (HWE); (O) “GG”, “GA”, and “AA” genotype frequency data of G20210A polymorphism in both cases and controls; (S) case-control studies. Articles were removed according to our specific exclusion criteria, which were duplicate studies, other disease or gene, cell or animal data, review or meta-analysis, meeting abstract, case report or trial, and lack of confirmed genotype data. When encountering disagreements, a discussion with another author (S. Zhao) took place for a final consensus.

### Data extraction

Three authors (Y. Zou, X. Ji, and Y. Liu) extracted the data from the eligible case-control studies. A form was utilized to summarize the characteristics, including the first author, publication year, country, ethnicity, genotype frequency, control source, genotyping assay, and sample size. When genotype frequency data was missing or unavailable, we tried to contact the author through an e-mail.

### Statistical analysis

Overall meta-analysis and subgroup analyses by three factors, including ethnicity, control source, and RVO type, were performed using Stata software (version 12.0, Stata Corporation, USA). In the heterogeneity test, a P value of Cochran's Q statistic larger than 0.1 or I^2^ value less than 50% indicated the existence of heterogeneity between studies, and a fixed-effect model was used in the association test (Mantel-Haenszel statistics). Four inheritance models, including A *vs* G (allele), A *vs* G (carrier), GA *vs* GG (heterozygote), and GA+AA *vs* GG (dominant), were utilized. The odds ratios (OR), 95% confidence intervals (CI), and P values of association tests were determined.

Begg's and Egger's tests were performed to assess the potential publication bias. P values less than 0.05 indicated the existence of potential publication bias. In addition, sensitivity analysis was performed to evaluate the stability of statistical results.

## Results

### Eligible case-control study selection

**Figure 1:**
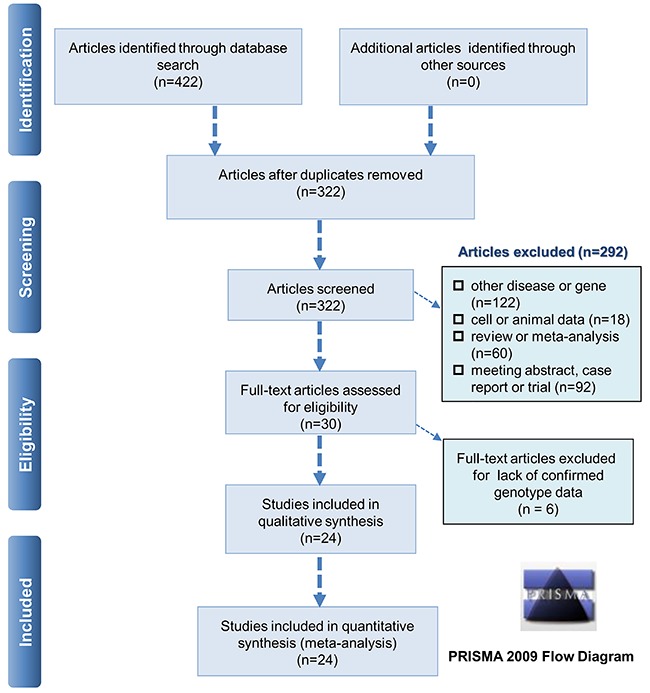
Flow diagram for identifying eligible case-control studies.

After the database search, we identified a total of 422 articles [PubMed (n=120), Embase (n=147), and WOS (n=155)] and removed the 100 duplicate articles. Then, we excluded another 292 improper articles, according to our exclusion criteria [other disease or gene (n=122), containing cell or animal data (n=18), review or meta-analysis (n=60), meeting abstract, case, or trial (n=92)]. Of the remaining 30 articles, six articles were excluded because no confirmed genotype data in both case and control groups were obtained. As a result, a total of 24 eligible case-control studies (3,6–28) were enrolled. Figure 1 shows the process for the selection of eligible studies, and Supplementary Table S2 summarizes the characteristics of case-control studies.

### Meta-analysis data

**Figure 2:**
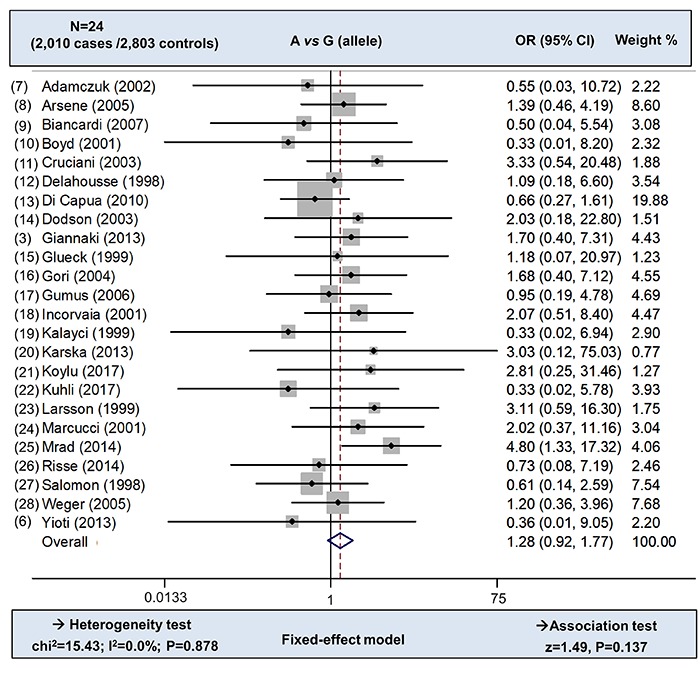
Overall meta-analysis under the A *vs* G (allele) model. OR: odds ratio; CI: confidence internal; N: study number.

There were a total of 24 case-control studies in the overall meta-analysis. The absence of heterogeneity (P value of the heterogeneity test >0.1, I^2^ =0.0%, Table 1) led to the application of Mantel-Haenszel statistics for the association test under the fixed-effect models. As shown in Table 2, we did not observe any statistical difference for the risk of RVO between cases and controls, under the inheritance models of A *vs* G (allele) (z=1.49, P*=*0.137), A *vs* G (carrier) (z=1.42, P=0.155), GA *vs* GG (z=1.50, P=0.135), and GA+AA *vs* GG (z=1.50, P=0.135). Figure 2 shows the forest plot data of overall meta-analysis under the A *vs* G (allele) model.

**Table 1:** Heterogeneity test and publication bias analysis.

Genetic models	Heterogeneity test				Model	Begg's test		Egger's test	
	N	I^2^	chi^2^	P		z	P	t	P
A *vs* G (allele)	24	0.0%	15.43	0.878	Fixed	1.17	0.244	-0.65	0.520
A *vs* G (carrier)	24	0.0%	14.42	0.914	Fixed	1.22	0.224	-0.68	0.504
GA *vs* GG	24	0.0%	15.93	0.858	Fixed	1.22	0.224	-0.66	0.515
GA + AA *vs* GG	24	0.0%	15.93	0.858	Fixed	1.22	0.224	-0.66	0.515

N: number of studies.

**Table 2:** Pooled analysis of the association between Prothrombin G20210A polymorphism and RVO risk.

Genetic model	Group	N	Association test			Sample size
			OR (95%CI)	P	z	
A *vs* G (allele)	Overall	24	1.28 (0.92 ∼ 1.77)	0.137	1.49	2,010 / 2,803
	Caucasian	22	1.17 (0.83 ∼ 1.67)	0.373	0.89	1,820 / 2,598
	PB	18	1.44 (0.99 ∼ 2.11)	0.058	1.90	1,214 / 2,053
	HB	5	0.74 (0.34 ∼ 1.63)	0.456	0.75	562 / 570
	CRVO	8	1.40 (0.76 ∼ 2.58)	0.283	1.07	549 / 980
	BRVO	6	1.05 (0.53 ∼ 2.07)	0.884	0.15	551 / 772
A *vs* G (carrier)	Overall	24	1.27 (0.91 ∼ 1.76)	0.155	1.42	2,010 / 2,803
	Caucasian	22	1.17 (0.82 ∼ 1.67)	0.383	0.87	1,820 / 2,598
	PB	18	1.43 (0.97 ∼ 2.10)	0.068	1.82	1,214 / 2,053
	HB	5	0.75 (0.34 ∼ 1.64)	0.467	0.73	562 / 570
	CRVO	8	1.38 (0.75 ∼ 2.56)	0.304	1.03	549 / 980
	BRVO	6	1.05 (0.53 ∼ 2.08)	0.888	0.14	551 / 772
GA *vs* GG	Overall	24	1.28 (0.93 ∼ 1.78)	0.135	1.50	2,010 / 2,803
	Caucasian	22	1.18 (0.82 ∼ 1.68)	0.372	0.89	1,820 / 2,598
	PB	18	1.45 (0.99 ∼ 2.13)	0.056	1.91	1,214 / 2,053
	HB	5	0.74 (0.34 ∼ 1.63)	0.453	0.75	562 / 570
	CRVO	8	1.40 (0.76 ∼ 2.60)	0.282	1.08	549 / 980
	BRVO	6	1.05 (0.53 ∼ 2.08)	0.887	0.14	551 / 772
GA + AA *vs* GG	Overall	24	1.28 (0.93 ∼ 1.78)	0.135	1.50	2,010 / 2,803
	Caucasian	22	1.18 (0.82 ∼ 1.68)	0.372	0.89	1,820 / 2,598
	PB	18	1.45 (0.99 ∼ 2.13)	0.056	1.91	1,214 / 2,053
	HB	5	0.74 (0.34 ∼ 1.63)	0.453	0.75	562 / 570
	CRVO	8	1.40 (0.76 ∼ 2.60)	0.282	1.08	549 / 980
	BRVO	6	1.05 (0.53 ∼ 2.08)	0.887	0.14	551 / 772

CRVO: central retinal vein occlusion; BRVO: branch retinal vein occlusion; PB: population-based control; N: number of studies; OR: odds ratio; CI: confidence interval.

### Subgroup analysis data

**Figure 3:**
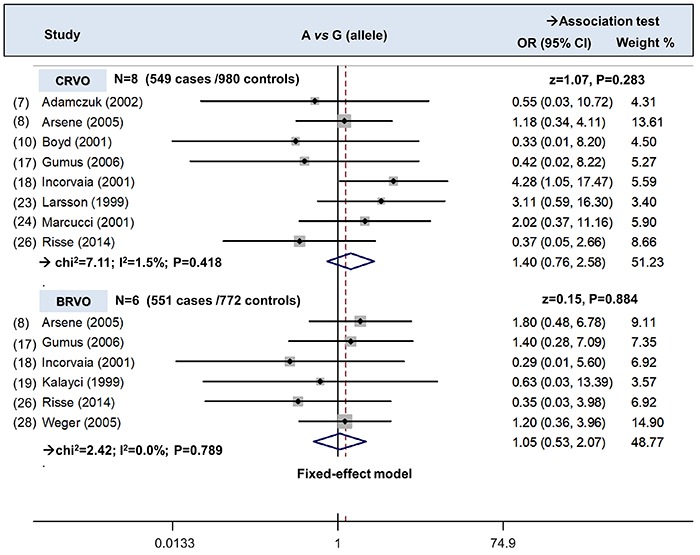
Subgroup analysis according to RVO type under the A *vs* G (allele) model. CRVO: central retinal vein occlusion; BRVO: branch retinal vein occlusion; OR: odds ratio; CI: confidence internal; N: study number.

Next, we performed the subgroup analyses by the factors of ethnicity (Caucasian), control source (population-based control; hospital-based control), and RVO type (BRVO or CRVO). As shown in Table 2, similar negative results were detected in the association test (all P>0.05). Figure 3 shows the forest plot data in subgroup analysis by RVO type under the A *vs* G (allele) model. These findings suggested that G20210A polymorphism within Prothrombin gene had no role influencing the risk of central or branch retinal vein occlusion in the Caucasian population.

### Publication bias and sensitivity analysis

**Figure 4:**
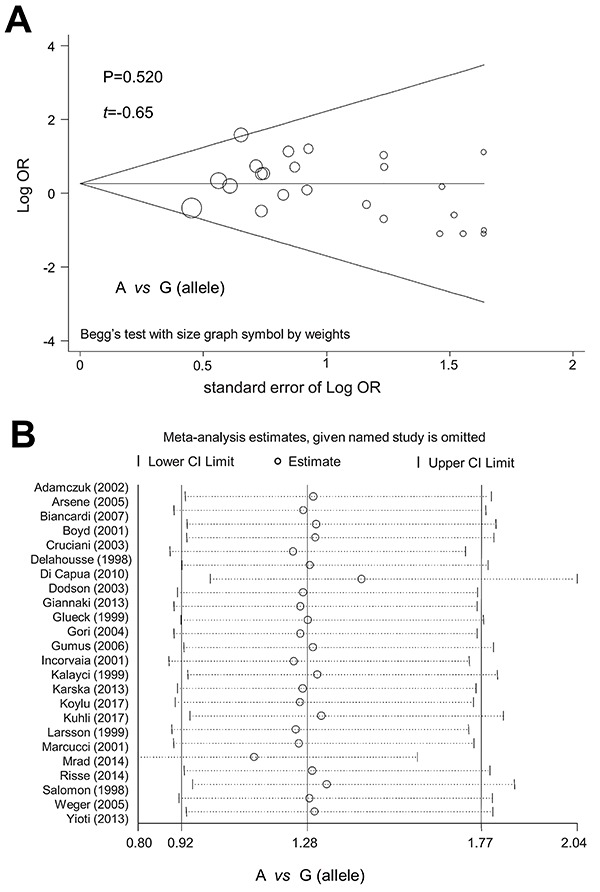
Publication bias plot of Begg's test (**A**) and sensitivity analysis data (**B**) under the A *vs* G (allele) model. See Figure 2 for reference numbers of articles cited. OR: odds ratio; CI: confidence internal.

We did not observe a significant publication bias in the above analyses, as P values in Begg's test and Egger's test were larger than 0.05 (Table 1). Figure 4A shows the Begg's publication bias plot under the A *vs* G (allele) model. Additionally, we observed a relatively stable conclusion through the sensitivity analysis (Figure 4B) for the allele model; data for other models are not shown.

## Discussion

Up to now, inconsistent conclusions on the association between Prothrombin G20210A polymorphism and RVO risk were reported. For instance, in Tunisia, Prothrombin G20210A polymorphism was reportedly linked to the risk of CRVO (P=0.007), rather than BRVO (P=0.09) (25). However, in Turkey, this polymorphism was reported not to be a risk factor of both CRVO and BRVO (19). One case-control study in Greece also reported a nonsignificant association between the polymorphism within Prothrombin gene and RVO susceptibility (6). Hence, it was meaningful to undertake the relevant meta-analysis for a comprehensive evaluation.

In 2005, Janssen et al. included six case-control studies (11 [Bibr B12] [Bibr B13],^23^,^24^,^27^,^29^,^30^) to perform the first meta-analysis, and provided the overall OR value of 1.6 and 95%CI of 0.8–3.2, but without the P value of association test (5). In 2013, Yioti et al. (6) conducted another meta-analysis with twenty studies (7–14,16–19,23,24,27,28,30–33) and reported a negative association between Prothrombin G20210A polymorphism and RVO susceptibility.

In order to enroll the maximum number of eligible case-control studies, we systematically searched the three on-line databases (PubMed, Embase, WOS) and applied a strict selection criteria. In comparison with the two prior reports, we ruled out one case-control study without Prothrombin G20210A mutation (32) and three studies without the confirmed genotype frequency data (30,31,33) in our updated quantitative meta-analysis. Moreover, a total of eight new case-control studies (3,6,15,20–22,25,26) were added. We conducted the overall meta-analysis and stratified analysis by three factors (ethnicity, control source, and RVO type), under the allele, carrier, heterozygote, and dominant models. Because the “AA” genotype frequency equaled to zero in each case-control study, we could not perform the meta-analysis under the homozygote (AA *vs* GG) and recessive (AA *vs* GG+GA) models. The same data was obtained in the heterozygote and dominant models. Although we utilized a new selection strategy and added some newly published case-control studies, no significant association between Prothrombin G20210A polymorphism and RVO risk was obtained in our updated pooling analysis.

Our sensitivity analyses data indicated the statistical robustness of pooling results while the heterogeneity Begg's and Egger's tests data supported the absence of high heterogeneity or publication bias. In spite of this, we should consider the existence of limitations within our meta-analysis. First, just like other meta-analyses, the statistical power of our pooling analysis was affected by the small number of enrolled studies. For instance, only one case-control study was enrolled in the Asian subgroup analysis (27). The negative association between Prothrombin G20210A polymorphism and the risk of RVO was mainly detected in Caucasian populations. More case-control studies in Asian and African populations are needed. Second, few publication regions, languages, or unpublished data may lead to the presence of selection bias. Third, due to the requirement of adequate genotype data, we only analyzed the genetic effect of one polymorphism within Prothrombin gene in our meta-analysis. We cannot exclude the potential role of other Prothrombin polymorphisms. Fourth, the joint effect of Prothrombin G20210A and other genetic polymorphisms, such as 4G/5G polymorphism of Plasminogen activator inhibitor-1 (*PAI-1*) gene and C677T (rs1801133) polymorphism of 5,10-methylenetetrahydrofolate reductase (*MTHFR*) gene, in the risk of ROV needs to be evaluated.

Prothrombin G20210A polymorphism was associated with enhanced susceptibility to venous thromboembolism (VTE), especially deep venous thrombosis and pulmonary embolism (34–36). Moreover, the positive association between Prothrombin G20210A polymorphism and the risk of VTE patients after arthroplasty surgery was observed in the Caucasian population, but not the Asian population (37). In addition, Prothrombin G20210A polymorphism was found to be a potential genetic marker for myocardial infarction of a Caucasian population in an age-related manner (38). However, our updated meta-analysis data did not support the association between Prothrombin G20210A polymorphism and the risk of RVO. It is well known that RVO is a type of complicated retinal vascular disease with multifactorial etiopathogenesis (1,39,40). The specific CRVO and BRVO types exhibit different risk factors, clinical features, and treatment methods (1,39,40). A series of factors, such as age, smoking, genetic variants, ethnic population, hypertension, diabetes, and hyperhomocysteinemia may be linked to the occurrence and development of RVO.

Taken together, our updated meta-analysis did not statistically support the genetic correlation between Prothrombin gene G20210A polymorphism and the risk of central or branch retinal vein occlusion. However, additional case-control studies in different populations are still needed.

## Supplementary material

Click here to view [pdf].
